# A Prediction Model Optimization Critiques through Centroid Clustering by Reducing the Sample Size, Integrating Statistical and Machine Learning Techniques for Wheat Productivity

**DOI:** 10.1155/2022/7271293

**Published:** 2022-03-11

**Authors:** Muhammad Islam, Farrukh Shehzad

**Affiliations:** Department of Statistics, The Islamia University of Bahawalpur, Bahawalpur, Pakistan

## Abstract

Machine learning algorithms are rapidly deploying and have made manifold breakthroughs in various fields. The optimization of algorithms got abundant attention of researchers being a core component for deploying the machine learning model (MLM) abled to learn the parameters in significant ways for the given data. Modeling crop productivity through innumerable agronomical constraints has become a crucial task for evolving sustainable agricultural policies. The cross-sectional datasets of 26430 (D1) crop-cut experiments are taken by 2nd-stage area frame sampling, collected from crop reporting service. This research is taken as follows: firstly three more effective numerical optimized datasets are generated (D1, D2, and D3) from D1 by taking the centroid points of features which decrease the sample size; secondly MLM is integrated with the traditional statistical models (TSMs) for multiple linear regression (MLR), and thirdly decision tree regression (DTR) and random forest regression (RFR) are deployed to get the optimized models able to predict the wheat productivity well with 75% datasets to train and 25% to test the model using the evaluation metrics (*R*^2^, RMSE), information criterion (AIC) with weights (AIC_W_), evidence ration (E.R), and decompositions of prediction error. The MLR outperformed for MLM than TSM. The performance capability of MLM and TSM got upswing for generated datasets. RFR got optimized and superperformed for D1, D2, D3, and D4. This study demonstrated strong evidences for deploying MLM for prediction of wheat productivity as an alternative of traditional statistical modeling.

## 1. Introduction

### 1.1. Significances, Motivations, and Objectives of the Study

Producing enough food for evolving population explosion has become the major concerns for the global world. Agriculture in aspect of core contributor in food production is ensuring to meet the sustainable food availability [1]. Food security has been considered as the foremost global threat, and therefore, it is essential to steer strategies to determine policies for future food security and sustainable food availability [2, 3]. Food and agricultural organization, international food policy research institute, and many other international organizations deem their great concerns on this converted threat to attain sustainable food availability [4–6]. Modeling crop productivity through innumerable agronomical constraints has become a crucial task to attain sustainable agriculture and for evolving effective agricultural strategies [7]. A precise crop model based on certain conditions is a foremost need of time to evoke to handle the prevailing food trepidations [8, 9]. Wheat being a 3rd largest food crop is playing a vital role for assuring the food supply in the world [4, 10–12]. Developing food prediction models, capable for true estimation of food availability, can assure veracious policy decision for managing national action plans for food security [13]. Pakistan stood 6th for wheat production, 8th for cultivated area under wheat crop, and 59th for wheat productivity [14]. Its exigent need of era is to develop accurate and precise wheat productivity model capable to predict the production on the reliable statistics which would help us to attain the assurance or nonassurance of future food demand [15]. Islam et al. [2] presented the study on the large datasets for building the statistical prediction model for the wheat productivity in Pakistan using hierarchical regression approach for selecting the features to address food security threat for the global concerns based on cross-sectional record. This study presented the tradition statistical modeling and introduced the theory of centroid clustering used to generate the three more datasets from the original datasets. Generated datasets enhanced the model prediction capability with the reduction of sample size. They applied different evaluation metrics, adjusted *R*^2^, Δ*R*^2^, MSE, and information criterion approaches such as Akaike information criteria (AIC), Schwarz information criterion (SIC), and weighted information criterion (Akaike weight “Wi”) with evidence ratio “E.R,” etc. The normality analysis and constant error variance are done by graphical presentation. The VIF is applied for multicollinearity, and nonconstant error variance is checked by Breusch–Pagan test which is developed in 1979 by Trevor Breusch and Adrian Pagan. The reliably analysis is performed by Cronbach's alpha test.

Machine learning algorithms widely develop and deploy rapidly and have made manifold breakthroughs in various fields. The advancement in science, technologies, and implementations of innumerable agronomical constraints in various fields of agriculture leads to immense volume of data [1, 8, 16]. The optimization of algorithms has become a significant part of machine learning and got abundant attention of researchers, and significance proficiency of numerical optimized algorithms of datasets affectedly influenced the machine learning model performance capability for the massive amount of data [17]. In this research, firstly the effective numerical optimized datasets are developed by taking the centroid points of features abled to enhance the machine learning model performance by decreasing the sample size, secondly machine learning models are integrated with the traditional statistical models, and thirdly different machine learning models are deployed to get the optimized models able to predict the wheat productivity well. This study designed to apply the supervise machine learning techniques, i.e., multiple linear regression model (MLRM), decision tree regression model (DTRM), and ensemble learning random forest regression model (RFRM) on the same datasets with the aim to enhance the model performance by reducing the sample size through centroid clustering. This study integrates the efficacies of machine learning algorithms with benchmark traditional statistical models for wheat productivity.

## 2. Material and Methods

### 2.1. Data Collection, Sampling Method, and Important Features Selection

Punjab is the 2nd largest province of Pakistan which accounted 76% share in total wheat cultivation area. The administrative setup of Punjab comprises upon nine divisions, thirty-six districts, and one hundred and forty-five tehsils. The 26,430 field of wheat crop-cut experiments (C.C.E) is taken from crop reporting service (CRS), Punjab, for the year comprised from 2016-17 to 2019-2020. The list frame sampling (LFS) technique using systematic random sampling (S_y_RS) in which complete village (sample unit) was selected as basic unit was remained in practice in CRS, but after 2018-19, 2nd-stage area frame sampling (AFS) is applied to select the sample for C.C.E [18].

(1) $$\begin{array}{c} \text{AFS}\left(P_{i}\right)=\frac{Z_{i}}{\sum_{i=1}^{N}Z_{i}}, \end{array}$$

where *Z*_*i*_ = cropped area of *i*^th^ village in *j*^th^ union councils of district, ∑_*i*=1_^*N*^*Z*_*i*_ = total crop area of village in *j*^th^ union councils of district, and *P*_*i*_ = probability of selecting the *i*^th^ village as sample. Qayyum and Shera [18] reported, at stage I, union councils are considered as population and village as sampling units using probability proportion to size (PPS), while at stage II, the selected sample village is considered as population and the land segment area is considered as sampling unit using the simple random sampling (SRS) techniques. The C.C.E is selected in land area segments. The wheat productivity with measuring scale munds/acre along with seven agronomical quantitative variables, i.e., fertilizer urea kg/acre, fertilizer DAP kg/acre, other fertilizers kg/acre, no. of water, seed quantity used kg/acre, no. of pest spry, no. of weeds spry, and eight binary categorical (0 for absence and 1 for presence) agronomical features, i.e., seed treatment, soil-type chikny loom, varieties adoption, harvest period April (1-20), planting in November, land irrigated, farmers' area >25 acres, and seed type, is used in the current study. Experiment is performed using *Python*'s key library called scikit-learn (Sklearn) by Jupyter Notebook as https://scikit-learn.org/stable/supervised_learning.html. Sklearn offers various prominent features for data processing, classification, clustering evaluation, and model selection. Model_selection is Sklearn method used for setting to analyze datasets and then using it on unseen datasets for evaluation purpose.

### 2.2. Supervised Machine Learning Technique

Machine learning is viewed as innovative extension of statistics capable of dealing with the massive datasets by adding the methods from computer science to the repertoire of statistics [19]. Machine learning is categorized as advanced tools applied for the prediction of agricultural production [20–23]. According to Jeong et al. [9], machine learning used latest process-based techniques as an alternative to traditional statistical modeling. Machine learning is viewed as assumption-free methods for correct data structure of model, and it is applied in complex projection concerns, i.e., function form for crop yield prediction [8, 24]. Arthur Samuel (1901–1990), a pioneer in artificial intelligence (AI), coined the term machine learning in 1959 as “Field of study that gives computers the capability to learn without being explicitly programmed” [25, 26]. The prominent layout of machine learning process is narrated as follows:

- Data gathering
- Data preparations
- Selection of machine learning model
- Data partitions into train and test split datasets
- Model evaluations for train model and for test model
- Hyperparametric tuning of machine learning models
- Deployment of ML model or prediction

#### 2.2.1. Multiple Linear Regression Models (MLRMs)

MLR is used to endeavor the relationship of feature with wheat productivity for prediction for both statistical and machine learning modeling as *Y*_*i*_=*X*_*i*_*β*_*i*_+*ε*, where *Y*_*i*_ = wheat productivity munds/acre, *X*_*j*_ = features, and *β*_*j*_ = features coefficients.

#### 2.2.2. Decision Tree Regression Model (DTRM)

**Figure 1:**
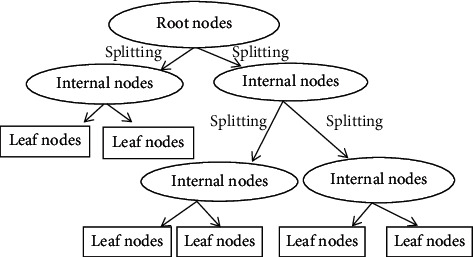
Structural flow of decision tree regression.

The decision tree regression model (DTRM) used the flowchart structure to predict the response. DTR-built internal node signifies a test, branches signify the outcome for test, and each leaf node signifies the final decision [27, 28]. In contemporary speech, leaf nodes reproduce the outcomes of prediction after getting hierarchal representation of leaf and branch structure for root-to-leaf direction. DTRM with depths ranging from 1 to 20 is plotted for training and test performance to determine the optimum DTRM capable to predict the wheat productivity well. The cross-sectional hyperparametric tuning is exercised using the GridSearchCV. The GridSearchCV is scikit-learn library applied to find out the optimum number for min_sample_split and max_depth (tree depth).  Figure 1 shows the structural flow for the decision tree model.

#### 2.2.3. Random Forest Regression Model (RFRM)

**Figure 2:**
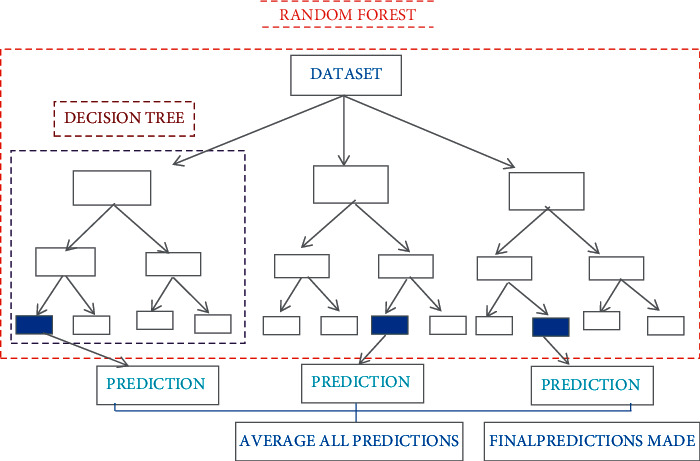
Structural flow of random forest regression.

The RFRM almost consists of the same set of hyperparameter tuning as DTRM except random forest (RF). RF used the additional randomness for the predicted made while growing the regression trees instead of pointing the important features to split the node. RFRM searches the best set of features and averaged multiple regression decision trees to avoid overfitting problem, and parameter no. of trees (*n*_sample) in the forest has been used which ranged from 10–100 [29, 30]. RFRM used precision to build up the forest random and search the best feature [31]. RFRM uses bootstrap aggregating for agricultural decision related to crop productivity prediction [21, 30, 31].  Figure 2 depicts the structural flow for RFR.

### 2.3. Preparation of Datasets

Data preprocessing is a technique used as a branch of data miming applied to search out accurate dataset from large dataset-based identifying, classification, clustering, and regression [32–34]. Three new datasets are generated from original 26,430 C.C.E by data preprocessing using centroid point clustering to increase the prediction interpretability and capability of models by reducing the sample size based at villages, tehsils, and district-level datasets [2].

(2) $$\begin{array}{c} {\bar{x}}_{i_{c_{m}}}=\frac{\sum_{j=1}^{N_{jn_{m}}}x_{i_{c_{m}}}}{N_{jn_{m}}},\\ {\bar{q}}_{i_{c_{m}}}=\frac{\sum_{j=1}^{N_{jn_{m}}}q_{i_{c_{m}}}}{N_{jn_{m}}}. \end{array}$$

For 1st subsets, *i* = 1, 2,…, 7 (quantitative variables), *j* = 1, 2,…, *N*_*jn*_*m*__ ( *j*^th^ observation of *i*^th^ predictors in *m*^th^ cluster (*m* = 1, 2, 3)), *N*_*jn*_*m*__ = total no. of *j*^th^ observation of *i*^th^ predictor in *m*^th^ cluster, and ${\bar{x}}_{i_{c_{m}}}$ = average of the *i*^th^ quantitative variable in *m*^th^ cluster. For 2nd subsets, *i* = 1, 2,…, 8 (categorical binary variables), *j* = 1, 2,…, *N*_*jn*_*m*__ ( *j*^th^ observation of the presence of *i*^th^ binary variable in *m*^th^ cluster (*m* = 1, 2, 3)), *N*_*jn*_*m*__ = total no. of *j*^th^ observation of *i*^th^ binary variable in *m*^th^ cluster, and ${\bar{q}}_{i_{c_{m}}}$ = proportion of the *i*^th^ binary predictor in *m*^th^ cluster. The original datasets (D_1_) comprise 26,430 rows/records/samples points of features, and the following three datasets are generated.

- Cluster-1 (D_2_) comprises 6034 rows/records/sample points taken by village centroid point of features
- Cluster-2 (D_3_) comprises 145 rows/records/sample points taken by tehsils centroid point of features
- Cluster-3 (D_4_) comprises 36 rows/records/sample points taken by district centroid point of features

### 2.4. Data Partition

Sklearn provides a way to generate accurate results abled to make true prediction, and for that, it is needed to train your model using train datasets and then test on unseen datasets using Sklearn train_test_split function. The train_test_split function is used for splitting a single dataset into two different subsets using random partitions called training subsets and testing subsets. The training subset is used to learn or to build model, and testing subset is used to evaluate the model performance for unseen datasets. For the current study, data partition is carried out using randomization train-test split and capability performance of models is investigated based on four types of datasets taking the 75% data as training subsets and 25% dataset for testing/validation subsets as follows.

- D_1_ consists of 19822 sample points as training subsets and 6608 subsets as testing subsets
- Cluster-1(D_2_) uses 4525 sample points as training and 1509 for testing subsets
- Cluster-2 (D_3_) uses 108 sample points as training and 37 for testing subsets
- Cluster-3(D_4_) uses 27 sample points as training subsets and 09 for testing subsets

### 2.5. Hyperparametric Tuning of Machine Learning Models

While applying the machine learning algorithms to predict the response variable (wheat productivity), the datasets split into two parts named training and testing datasets (Section 2.4). Two types of error are reported in prediction of response using machine algorithms [35], the error reported during training phase is called training error or bias, and this error is measured from overall observed data samples in the training phase, while the out-of-sample error (generalization error) measures the expected error on testing phase or in unseen datasets called variance. Both the underfit (high bias and high variance) and overfit (low bias and high variance) algorithms mislead the machine learning model prediction capability, and the bias-variance trade-off is common property in application of machine learning model building. The decomposition of prediction error is comprised as the sum of three components, bias, variance, and irreducible error [25, 36]. The mathematical illustration of bias and variance is presented as the target variable (wheat yield) is going to be predicted by machine learning model taking the covariates (15 features) by the relation as *y* = *g*(*x*) + *e* where “*e*” is supposed to be the error term fallow normality. Using machine learning modeling technique, the estimated model of *g*(*x*) is $\hat{g}\left(x\right)$ and the expected squared prediction error at “*x*” is found as follows:

(3) $$\begin{array}{c} P\text{.E}\left(x\right)=E\left[y-g{\left(x\right)}^{2}\right]. \end{array}$$

Prediction error is decomposed into categories as bias and variance components as follows:

(4) $$\begin{array}{c} E{\left[y-\hat{g}\left(x\right)\right]}^{2}={\left[E\left\{\hat{g}\left(x\right)\right\}-g\left(x\right)\right]}^{2}+E\left[{\left\{\hat{g}\left(x\right)-E\left[\hat{g}\left(x\right)\right]\right\}}^{2}\right]+\sigma_{e},\\ P\text{.E}\left(x\right)=\text{Var}\left[\hat{g}\left(x\right)\right]+{\left[\text{Bias}\left\{\hat{g}\left(x\right)\right\}\right]}^{2}+\text{Var}\left(e\right),\\ \text{prediction error}=\text{variance}+{\text{bias}}^{2}+\text{irreducible error term}. \end{array}$$

**Figure 3:**
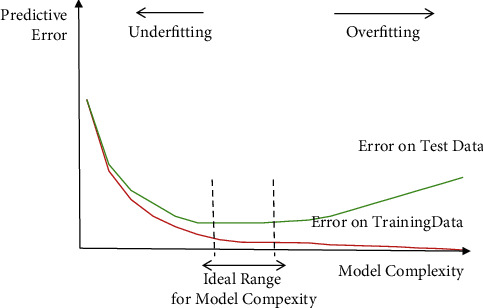
Structure of MLM complexity for over- and underfitting.

That irreducible error term may be known as noise term which exists in the true relationship between the feature and response in model prediction and in machine learning model; the aim is to decrease both the bias and variance terms. However, in machine learning model prediction, there exists a bias-variance trade-off and the optimum model complexity means a situation where the model predicted well with low variance and low bias and is free from overfit and underfit model [37].  Figure 3 elaborates the condition of overfitting and underfitting at lower and higher model complexity, while at ideal range of model complexity, the MLM predicted well.

### 2.6. Evaluation Metrics and Information Criterion

The evaluation metrics using the performance score (*R2*) and root mean square error (RMSE) are applied to measure the accuracies of regression models. Lower the value of RMSE and higher the performance score lead to support the good fit.

(5) $$\begin{array}{c} \text{RMSE}=\sqrt{\frac{\sum_{i}^{n}{\left(y_{i}-{\bar{y}}_{i}\right)}^{2}}{n}},\\ R^{2}=\frac{\sum {\left(\hat{Y}-\bar{Y}\right)}^{2}}{\sum {\left(Y-\bar{Y}\right)}^{2}}. \end{array}$$

#### 2.6.1. Akaike Information Criterion, AIC Weights, Evidence Ratio, and Reliability Analysis

The Akaike information criterion (AIC) using the log-likelihood functions with simple penalties is applied to determine the theoretical and logical relevance of the predictors to the response and their statistical significance in model. Lower the value of AIC leads to conclude that the fitted regression model is good [38–40].

(6) $$\begin{array}{c} \text{AIC}=e^{2k/n}\frac{\sum {\hat{u}}_{i}^{2}}{n}\\ =e^{2k/n}\frac{\text{RSS}}{n}, \end{array}$$

where *k* = no. of features and intercept, *n* = sample size, and 2*k*/*n* = penalty factor.

One of the key objectives of driving the AIC is to determine the range of models with their relative AIC value. For comparing the multiple models, we can measure how much better the best candidate model is to be compared with next best models, and the easiest way to determine the comparison is to measure the change in of AIC values for the best model with the *i*^th^ other models ΔAIC_*i*_ = AIC_*i*_ − AIC_min_. ΔAIC_*i*_ is also used to measure the relative strengths of best models with other models. ΔAIC_*i*_ is used to determine the level of empirical support of model comparisons for quick strength of evidence, and lower the difference leads to support the model. Burnham and Anderson [41] defined the evidence ratio “E.R” used to compare the efficiencies of various models and depicted the measure of how much more likely the best model is than other models [42].

(7) $$\begin{array}{c} \text{ER}=\frac{\exp\left(-\left(1/2\right)\Delta_{\text{best}}\right)}{\exp\left(-\left(1/2\right)\Delta_{i}\right)}=\frac{{\text{Weight}}_{\text{best}}\left(\text{AIC}\right)}{W_{j}\left(\text{AIC}\right)}. \end{array}$$

Akaike weight is used to determine the probability of model having good prediction capability or not to predict the wheat productivity and summing to unity [∑*W*_*i*_(AIC)=1]. The higher weights lead to model having relatively good prediction capability and vice versa [38, 43]. Cronbach's alpha “*α*” and reliability analysis are applied to determine the degree of consistency and relevance of predictors with reference to the measure of response [44, 45].

(8) $$\begin{array}{c} W_{i}\left(\text{AIC}\right)=\frac{\exp\left\{-\left(1/2\right)\Delta_{i}\left(\text{AIC}\right)\right\}}{\sum_{k=1}^{n}\exp\left\{-\left(1/2\right)\Delta_{i}\left(\text{AIC}\right)\right\}},\\ \text{Corn bach's }\alpha ==\frac{k}{k-1}\left(1-\frac{\sum s_{i}^{2}}{s_{T}^{2}}\right), \end{array}$$

where *k* = no. of items, *s*_*i*_^2^ = variance of *i*^th^ item, and *s*_*T*_^2^ = aggregate item variance.

Reliability coefficient ranging from 0 to 1 and its values near to 0 indicate poor reliability while near to 1 depict strong reliability. The prediction capabilities of models are integrated by using the four different sample size datasets generated through centroid clustering scheme. This study integrates the efficacies of machine learning models with benchmark traditional statistical models to select the most optimum model that follows the evaluation metrics and information criteria.

## 3. Data Analysis

### 3.1. Importance of Agronomical Features and Reliability of Datasets

**Figure 4:**
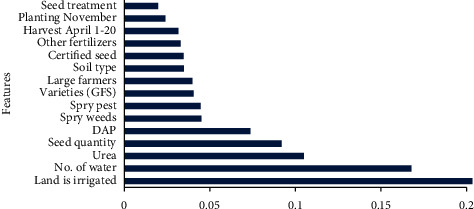
Feature importance for D1.

**Figure 5:**
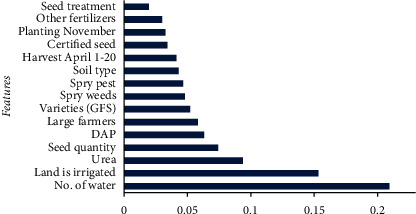
Feature importance for D2.

**Figure 6:**
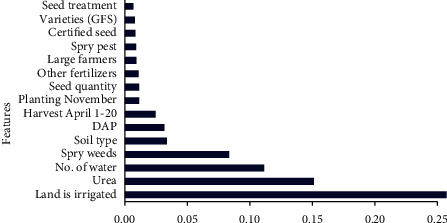
Feature importance for D3.

**Figure 7:**
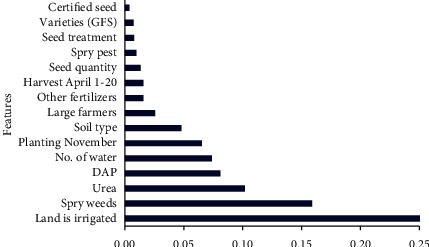
Feature importance for D4.

Feature importance refers to techniques that ascribe importance score to input variables which are useful to investigate that how useful the features are to predict the response. Feature importance scores provide the view insight datasets as well as inside the model and improved the efficiency, predictability, and effectiveness of a predictive machine learning model. Before deployment of machine approaches to different datasets, the variations of agronomical features prevailed in simultaneous order for the importance of usefulness in the current study are particularized in Figure 4 for D1, Figure 5 for D2, Figure 6 for D3, and Figure 7 for D4.  Table 1 shows the values of Cronbach's alpha for the reliability measure and reports the reliability coefficients as 0.35 for D1, 0.39 for D2, 0.63 for D3, and 0.64 for D4. The reliability of datasets has become strong and strongest as we advanced from D1 to D4.

### 3.2. Performance Measures of Multiple Linear Regression Models

The performance for the prediction capability of multiple linear regression for the generated different size datasets is evaluated and integrated for both the traditional statistical models and machine learning approaches.

### 3.3. Machine Learning Models

Multiple linear regression models (MLRMs) are constructed using the machine learning approach and integrated with benchmark traditional statistical models. For MLM, Table 2 shows the performance score 0.266, 0.289, 0.838, and 0.932 for the training datasets and 0.264, 0.285, 0.834, and 0.655 for testing/validated datasets, respectively, for D1, D2, D3, and D4. The *R*^2^ has become strong and strongest as we advance from D1 to D4 for train datasets (*R*_Dtrain(*i*)_^2^ < *R*_Dtrain(*i*+1)_^2^) and de novo the same for test data except for D4. The RMSE found 9.14 and 9.21 for D1, 7.65 and 8.09 for D2, 3.15 and 3.34 for D3, 1.95 and 3.31 for D4, respectively, for train and test models. The RMSE decreases as we advanced from D1 to D4 (RMSE_*D*(*i*)_ > RMSE_*D*(*i*+1)_) for both train and test datasets. The model is train and deployed for the training datasets using 75% train subsets. The D4 shows lowest AIC as 1.62 with highest Akaike weights (AIC_W_) as 0.45 followed by AIC as 2.43 and AIC_W_ as 0.30 for D3, AIC as 4.07 and AIC_W_ as 0.13 for D2, and AIC as 4.43 and AIC_W_ as 0.11 for D1. The Akaike weights are increasing (AIC_*w*(*i*)_ < AIC_*w*(*i*+1)_), and AIC is decreasing (AIC_(*i*)_ > AIC_(*i*+1)_) as we advance from D1 to D4. The evidence ratio justifies the results as D4 model is 4.06, 3.41, and 1.50 more likely to D1, D2, and D3 models, respectively.

#### 3.3.1. Integrating Machine Learning and Traditions Statistics Modeling for MLR

**Figure 8:**
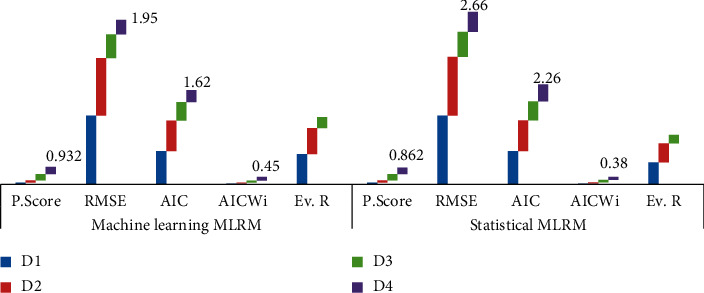
Integrating/comparisons of machine learning and statistical models.

Table 2 shows the comparisons of model performance for MLM with benchmarks TSM. For TSM, the performance score is found as 0.265, 0.287, 0.823, and 0.862 and RMSE as 9.17, 7.77, 3.35, and 2.66, respectively, for D1, D2, D3, and D4. It is evident that the highest values of performance score and lowest value of RMSE are found for MLM comparing with benchmark TSM as we advanced from D1 to D4 (*R*_TSM_^2^ < *R*_MLM_^2^, RMSE_TSM_ > RMSE_MLM_). The lowest value of AIC is obtained from MLM comparing with TSM for all the datasets as AIC_TSM_ > AIC_MLM_. The AIC weight reported 0.45 for MLM, while it is 0.38 for TSM for D4 which elaborated as MLM has high probability for selecting the best model. The evidence ratio for TSM based on D4 is 2.96, 2.51, and 1.14 more likely to D1, D2, and D3 and integrated that E.R is found better in MLM comparing with TSM for all datasets (E.*R*_TSM_ < E.R_MLM_). All the performance measure optimized well in ML models clarified that MLM has good prediction capability for prediction of the wheat productivity based on agronomical features.  Figure 8 clarifies that the graphical relations exist for learning points of the models for evaluation metrics and information criterion for both MLM and TSM and shows that machine learning performed well for all the datasets and D4 optimized the machine learning multiple regression models.

### 3.4. Decision Tree and Random Forest Regression Models

The machine learning models are trained and deployed for multiple linear regression models, and predicted well is further trained and deployed for the important and most prominent machine algorithms, i.e., decision tree regression models (DTRMs) and random forest regression models (RFRMs) with the aim to get the most optimized models able to predict the wheat productive well using 75% data to learn the model and 25% as validated datasets to evaluate the model capability on unseen datasets.

#### 3.4.1. Hyperparametric Tuning of DTRM and RFRM

**Figure 9:**
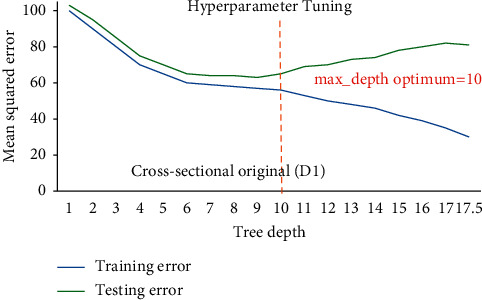
Hyperparameter tuning of DTR for D1.

**Figure 10:**
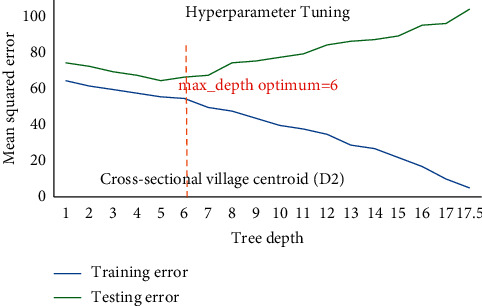
Hyperparameter tuning of DTR for D2.

**Figure 11:**
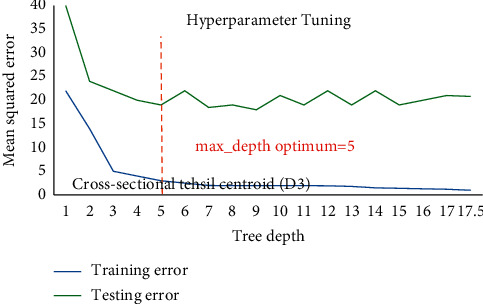
Hyperparametric tuning of DTR for D3.

**Figure 12:**
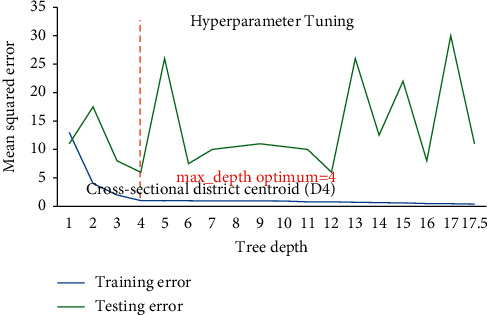
Hyperparametric tuning of DTR for D4.

Hackeling [46] reported hyperparametric tuning of DTRM models applied to avoid over and underfitting using the scikit-learn's library GridSearchCV to find out the optimum value of min_sample_split and max_depth (tree depth).  Figure 9 shows DTR for D1 having 19822 samples point for training and 6608 sample points for testing phase and illustrates that at lower model complexity the model is underfit (high bias and high variance) and the error curve for testing set raises again after tree depth 10 which leads to overfit the model, while for Figure 10, DTR for D2 has 4525 sample points for training and 1509 sample points for testing phase, and the same prevails after tree depth 06, indicating that optimum hyperparameter for tree depth is found 10 and 06 for DTR model based on D1 and D2. The tree depth values got optimized at 05 and 04 for models based on D3 having 108 sample points for training and 37 sample points for testing phase and D4 having 27 sample points for training and 09 sample points for testing phase (Figures 11 and 12). The min_sample_split value found optimized at 29, 28, 6, and 2, respectively, for D1, D2, D3, and D4. The RFR and DTR consist of the same set hyperparameters except random forest called no. of trees in the forest (*n*_sample) and its default value ranged from 10-100. The D1 optimized at no. of tree 10, D2 and D3 at no. of tree 50, and D4 optimized at no. of tree 100 for the prediction model for wheat productivity.

#### 3.4.2. Decision Tree Regression Models

For the DTRM, Table 3 shows the performance score and RMSE as 0.364, 0.366, 0.940, and 0.987 and 8.51, 7.22, 1.92, and 0.828 for train models, while for test model the performance scores are 0.323, 0.331, 0.731, and 0.741 and RMSEs are 8.82, 7.82, 4.26, and 2.87. *R*^2^ is increasing, and RMSE is decreasing (*R*_*D*(*i*)_^2^ < *R*_*D*(*i*+1)_^2^, RMSE_*D*(*i*)_ > RMSE_*D*(*i*+1)_) for train and test models as we advanced from D1 to D4. The DTR model is trained and deployed for the training datasets using 75% train subsets. The AIC reported diminishing trend as 4.28, 3.96, 1.44, and 0.29 for D1 to D4 (AIC_(*i*)_ > AIC_(*i*+1)_). The AIC_W_ of models based on D4 is highest with probability 0.54 followed by D3 = 0.30, D2 = 0.13, and D1 = 0.07 (AIC_w(*i*)_ < AIC_w(*i*+1)_). The E.R values of DTR models show that the model learns from D4 is 7.37, 6.27, and 1.78 more likely to models learns from D1, D2, and D3.

#### 3.4.3. Random Forest Regression Models

For the RFR, Table 3 shows the performance score and RMSE as 0.380, 0.388, 0.948, and 0.973 and 8.40, 7.09, 1.78, and 1.23 for train sets, while the performance score and RMSE is reported as 0.345, 0.362, 0.786, and 0.877 and 8.68, 7.64, 3.79, and 1.97 for test models. *R*^2^ shows the increasing, and RMSE shows the diminishing relation as we advanced from D1 to D4 (*R*_*D*(*i*)_^2^ < *R*_*D*(*i*+1)_^2^, RMSE_*D*(*i*)_ > RMSE_*D*(*i*+1)_). The RFR model is trained and deployed for the training datasets using 75% train subsets. The AIC reported diminishing trend 4.26, 3.92, 2.18, and 0.70 with increasing AIC_W_ as 0.09, 0.11, 0.26, and 0.54 for D1, D2, D3, and D4 models (AIC_w(*i*)_ < AIC_w(*i*+1)_ and AIC_(*i*)_ > AIC_(*i*+1)_). The highest values of AIC weight reported from model learn from D4 followed by models learn from D3, D2, and D1. The E.R values of RFR models show that the models learn from D4 and are 5.92, 5.0, and 2.10 more likely to models learn from D1, D2, and D3.

### 3.5. Comparative Quantification of Machine Learning Models for Different Datasets

Section 3.3.1 depicts that machine learning performed well comparing with traditional statistical approaches for multiple regression models.  Section 3.3 presents models further trained and deployed for machine learning algorithms, i.e., decision tree regression models (DTRMs) and random forest regression models (RFRMs) with the aim to get the most optimized models able to predict the wheat productive well.

**Figure 13:**
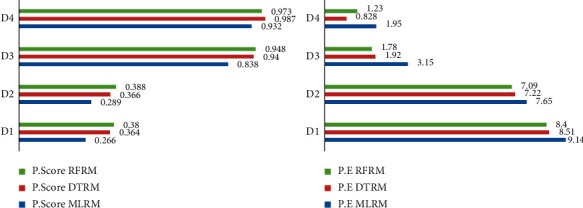
The performance measure of MLMLR, DTR, and RFR machine learning models.

**Figure 14:**
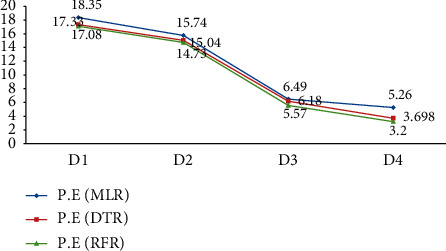
Integrating decomposition prediction error for MLR, DTR and RFR.

In Tables 2 and 3 and Figure 13, the performance score of RFR models is reported well for all training and testing datasets followed by DTR and MLR for D1 and D2. The performance score of RFR is found high for D3 training set, while little bit variation is found for testing sets, and for D4 all models show performance above 90% for training sets and only RFR approach to 0.877% for testing/validation datasets, while DTR has 0.741 and MLR has 0.655. The RMSE of RFRM reported low for D1 and D2 for train and de novo the same for test models. The RFRM shows good for D3 train models, while MLR supersedes on slight extent for test models. The DTR performed well for D4 train model, while for test model RFR supersedes the DTR. The MLM train and deployed for training datasets revealed the relation as (*R*_MLRM_^2^ < *R*_DTRM_^2^ < *R*_RFRM_^2^), (RMSE_MLRM_ > RMSE_DTRM_ > RMSE_RFRM_)for D1, D2 and D3, while for D4, all models show high performance score as MLR = 0 .932, DTR = 0.987 and RFR = 0.973. Data preprocessing optimized the model predictability well for all datasets as all models upswing the performance from original datasets (D1) to generated datasets (D2, D3, D4) for MLM. In Figure 14, learning curves (L.E) demonstrate the comparison for decomposition of prediction error (P.E), and it is validated that RFRM revealed lower prediction error simultaneously for D1, D2, D3, and D4 prediction models as 17.08, 14.73, 5.57, and 3.2 followed by DTR as 17.33, 15.04, 6.18, and 3.698 and MLR 18.35, 15.74, 6.49, and 5.26.

(P.EMLRMDi) >(P.EDTRMDi) > (P.ERFRMDi). RFRM revealed good performance score and bottommost decomposition prediction error as we advanced from D1 to D4. RFRM successfully predicted the wheat productivity when compared against other models using the original and generated datasets.

## 4. Conclusions

This study integrated the efficacies of machine learning regression algorithms using multiple linear regression models (MLRMs), decision tree regression models (DTRMs), and random forest regression models (RFRMs) with benchmark traditional statistical models to converge the optimization capability of prediction models for wheat productivity. The original dataset of 26430 (D1) crop-cut experiment along with fifteen features is collected from the crop reporting service. The 2nd-stage area frame sampling is applied to select the sample. The new approach of centroid clustering scheme is introduced which can enhance the model performance by reducing the sample size. Three more datasets are generated to optimize the model performance for both the machine learning models (MLMs) and traditional statistical models (TSMs). The generated datasets comprise from 6034, 145, and 36 sample points generated from village, tehsil, and district-level centroid clusters. The 75% dataset is used as training and 25% as testing subsets. Evaluation metrics approach (*R*^2^, RMSE), Akaike information criterion (AIC) with weights (AIC_W_), evidence ration (E.R), reliability analysis, and decomposition prediction error (P.E) are applied to compare the performance of models. The performance score (P.S) increased, while the RMSE and AIC decreased for both MLM and TSM as we advanced from D1 to D4 for MLRM. The P.S and E.R reported high (E.R_TSM_ < E.R_MLM_ & *R*_TSM_^2^ < *R*_MLM_^2^), while RMSE and AIC reported low (RMSE_TSM_ > RMSE_MLM_ & AIC_TSM_ > AIC_MLM_) for MLM comparing with benchmark TSM as we proceed from D1 to D4 for MLRM. The MLM based on MLRM has good prediction capability for all the datasets, and D4 optimized the MLM. The MLM trained and deployed for MLRM is further trained and deployed for DTRM and RFRM with the aim to get the most optimized model. RFRM revealed good P.S, bottommost P.E for all the datasets. The RFRM successfully predicted the wheat productivity followed by DTRM and MLRM for D1, D2, D3, and D4. It is demonstrated that machine learning models provide superior performance by centroid clustering even for sample size as we advanced from D1 to D4. This study demonstrated strong evidences for the implementation of machine learning models as an alternative of traditional statistical models for future research direction and correct policy decisions regarding wheat productivity. The advancement in science, technologies, and implementations of innumerable agronomical constraints in various fields of agriculture leads to immense volume of data, and this study provides the detailed hierarchy of centroid clustering which leads to increase the model performance by reducing the sample size. This hierarchy of centroid clustering could also be extended to multistage centroid clustering for future research, and it could also be applied for all supervised machine learning algorithms to enhance the model performances.

## Figures and Tables

**Table 1:** Cronbach's alpha reliability coefficients for various datasets.

Cronbach's alpha coefficients	D1	D2	D3	D4
	0.35	0.39	0.63	0.64

**Table 2:** Integrating machine learning and tradition statistics modeling for MLR.

Datasets	Machine learning MLRM						Statistical MLRM					
	P.Score	RMSE	AIC	ΔAIC_*i*_	AIC_Wi_	E.R	P.Score	RMSE	AIC	ΔAIC_i_	AIC_Wi_	E.R
D_1_	0.266 (0.264)	9.14 (9.21)	4.43	2.80	0.11	4.06	0.265	9.17	4.43	2.17	0.13	2.96
D_2_	0.289 (0.285)	7.65 (8.09)	4.07	2.45	0.13	3.41	0.287	7.77	4.10	1.84	0.15	2.51
D_3_	0.838 (0.834)	3.15 (3.34)	2.43	0.81	0.30	1.50	0.823	3.35	2.52	0.26	0.34	1.14
D_4_	0.932 (0.655)	1.95 (3.31)	1.62		0.45		0.862	2.66	2.26		0.38	

Testing dataset values are shown in parenthesis.

**Table 3:** Integrating the DTR and RFR with evaluation metric and information criteria.

Datasets	Machine learning DTR						Machine learning RFR					
	P.Score	RMSE	AIC	ΔAIC_*i*_	AIC_Wi_	E.R	P.Score	RMSE	AIC	ΔAIC_i_	AIC_Wi_	E.R
D_1_	0.364 (0.323)	8.51 (8.82)	4.28	4.00	0.07	7.37	0.380 (0.345)	8.40 (8.68)	4.26	3.56	0.09	5.92
D_2_	0.366 (0.331)	7.22 (7.82)	3.96	3.67	0.13	6.27	0.388 (0.362)	7.09 (7.64)	3.92	3.22	0.11	5.00
D_3_	0.940 (0.731)	1.92 (4.26)	1.44	1.15	0.30	1.78	0.948 (0.786)	1.78 (3.79)	2.18	1.48	0.26	2.10
D_4_	0.987 (0.741)	0.828 (2.87)	0.29		0.54		0.973 (0.877)	1.23 (1.97)	0.70		0.54	

Testing dataset values are shown in parenthesis.

## Data Availability

The cross-sectional original datasets and generated datasets used to support the findings of this study are available from the corresponding author upon request.

## References

[1] Elavarasan D., Vincent P. D. R. (2021). A reinforced random forest model for enhanced crop yield prediction by integrating agrarian parameters. *Journal of Ambient Intelligence and Humanized Computing*.

[2] Islam M., Shehzad F., Omar M. (2021). Modeling wheat productivity using hierarchical regression: a way to address food security concerns. *Ilköğretim Online*.

[3] Shoaib S. A., Khan M. Z. K., Sultana N., Mahmood T. H. (2021). Quantifying uncertainty in food security modeling. *Agriculture*.

[4] Islam M. (2017). Factors affecting major food crops production a case study of District Bahawalpur.

[5] Nelson G. C., Rosegrant M. W., Palazzo A. (2010). *Food Security, Farming, and Climate Change to 2050: Scenarios, Results, Policy Options*.

[6] Timmer C. P. (2014). Food security in asia and the pacific: the rapidly changing role of rice. *Asia & the Pacific Policy Studies*.

[7] Elavarasan D., Vincent D. R. (2020). Reinforced XGBoost machine learning model for sustainable intelligent agrarian applications. *Journal of Intelligent and Fuzzy Systems*.

[8] Elavarasan D., Vincent P. D. R. (2021). Fuzzy deep learning-based crop yield prediction model for sustainable agronomical frameworks. *Neural Computing & Applications*.

[9] Jeong J. H., Resop J. P., Mueller N. D. (2016). Random forests for global and regional crop yield predictions. *PLoS One*.

[10] Enghiad A. (2015). *Examining the Response of World Wheat Prices to Climatic and Market Dynamics*.

[11] Kiss I. (2011). Significance of wheat production in world economy and position of Hungary in it. *APSTRACT: Applied Studies in Agribusiness and Commerce*.

[12] Ramesh C. (2009). Challenges to ensuring food security through wheat. *CAB reviews: Perspectives in agriculture, veterinary science, nutrition and natural resources*.

[13] Nosratabadi S., Ardabili S., Lakner Z., Mako C., Mosavi A. (2021). Prediction of food production using machine learning algorithms of multilayer perceptron and ANFIS. *Agriculture*.

[14] Zulfiqar F., Hussain A. (2014). Forecasting wheat production gaps to assess the state of future food security in Pakistan. *Journal of Food and Nutritional Disorders*.

[15] Sharma I., Tyagi B., Singh G., Venkatesh K., Gupta O. (2015). Enhancing wheat production—a global perspective. *Indian Journal of Agricultural Sciences*.

[16] Elavarasan D., Vincent P. D. (2020b). Crop yield prediction using deep reinforcement learning model for sustainable agrarian applications. *IEEE Access*.

[17] Sun S., Cao Z., Zhu H., Zhao J. (2019). A survey of optimization methods from a machine learning perspective. *IEEE Transactions on Cybernetics*.

[18] Qayyum A., Shera H. M. J. (2019). Method of area frame sampling using probability proportional to size sampling technique for crops’ surveys: a case study in Pakistan. *Journal of Experimental Agriculture International*.

[19] Cielen D., Meysman A., Ali M. (2016). *Introducing Data Science: Big Data, Machine Learning, and More, Using Python Tools*.

[20] Alagurajan M., Vijayakumaran C. (2020). ML methods for crop yield prediction and estimation: an exploration.

[21] Elavarasan D., Vincent D. R., Sharma V., Zomaya A. Y., Srinivasan K. (2018). Forecasting yield by integrating agrarian factors and machine learning models: a survey. *Computers and Electronics in Agriculture*.

[22] Mishra S., Mishra D., Santra G. H. (2016). Applications of machine learning techniques in agricultural crop production: a review paper. *Indian Journal of Science and Technology*.

[23] Yadav N. (2020). Machine learning in agriculture: techniques and applications.

[24] Priya P., Muthaiah U., Balamurugan M. (2018). Predicting yield of the crop using machine learning algorithm. *International Journal of Engineering Sciences & Research Technology*.

[25] Dangeti P. (2017). *Statistics for Machine Learning*.

[26] McCarthy J., Feigenbaum E. A. (1990). In memoriam: Arthur Samuel: pioneer in machine learning. *AI Magazine*.

[27] Quinlan J. R. (1987). Simplifying decision trees. *International Journal of Man-Machine Studies*.

[28] Utgoff P. E. (1989). Incremental induction of decision trees. *Machine Learning*.

[29] Alif A. A., Shukanya I. F., Afee T. N. (2018). *Crop Prediction Based on Geographical and Climatic Data Using Machine Learning and Deep Learning*.

[30] Breiman L. (2001). Random forests. *Machine Learning*.

[31] Venishetty S. V. (2019). Machine learning approach for forecasting the sales of truck components.

[32] Elavarasan D., Vincent D. R. (2017). Effective mining approach to produce quality search results using proposed approach. *International Journal of Intelligent Engineering and Systems*.

[33] Han J., Kamber M., Pei J. (2011). Data mining concepts and techniques. *The Morgan Kaufmann Series in Data Management Systems*.

[34] Rahman A. (2019). Statistics-based data preprocessing methods and machine learning algorithms for big data analysis. *International Journal of Artificial Intelligence*.

[35] Igual L., Seguí S. (2017). *Introduction to Data Science*.

[36] Geurts P. (2009). Bias vs variance decomposition for regression and classification. *Data Mining and Knowledge Discovery Handbook*.

[37] Jain A. (2016). Complete guide to parameter tuning in XGBoost (with codes in Python). https://www.analyticsvidhya.com/blog/2016/03/completeguide-parameter-tuning-xgboost-with-codes-python.

[38] Banks H. T., Joyner M. L. (2017). AIC under the framework of least squares estimation. *Applied Mathematics Letters*.

[39] Dziak J. J., Coffman D. L., Lanza S. T., Li R. (2012). Sensitivity and specificity of information criteria.

[40] Gujarati D. N. (2003). *Basic Econometrics*.

[41] Burnham K., Anderson D. R. (2002). *Model Selection and Multimodel Inference*.

[42] Symonds M. R., Moussalli A. (2011). A brief guide to model selection, multimodel inference and model averaging in behavioural ecology using Akaike’s information criterion. *Behavioral Ecology and Sociobiology*.

[43] Wagenmakers E.-J., Farrell S. (2004). AIC model selection using Akaike weights. *Psychonomic Bulletin & Review*.

[44] Bland J. M., Altman D. G. (1997). Statistics notes: Cronbach’s alpha. *BMJ*.

[45] Sekaran U., Bougie R. (2016). *Research Methods for Business: A Skill Building Approach*.

[46] Hackeling G. (2017). *Mastering Machine Learning with Scikit-Learn*.

